# Florida Obsessive‐Compulsive Inventory and Children's Florida Obsessive Compulsive Inventory: A reliability generalization meta‐analysis

**DOI:** 10.1002/jclp.23416

**Published:** 2022-07-18

**Authors:** Alejandro Sandoval‐Lentisco, Rubén López‐Nicolás, José Antonio López‐López, Julio Sánchez‐Meca

**Affiliations:** ^1^ Department of Basic Psychology and Methodology University of Murcia Murcia Spain

**Keywords:** Cronbach's *α* coefficient, Florida Obsessive Compulsive Inventory, meta‐analysis, obsessive‐compulsive disorder, reliability generalization

## Abstract

**Background:**

The Florida Obsessive Compulsive Inventory (FOCI) and its pediatric version, the Children's Florida Obsessive Compulsive Inventory (C‐FOCI), are instruments for evaluating obsessive‐compulsive symptomatology.

**Method:**

A reliability generalization meta‐analysis was conducted to estimate an average reliability of the scores and to identify study characteristics that explained the heterogeneity among scores. Using Kuder–Richardson 20 (KR‐20) and Cronbach's *α*, a total of 23 and 20 independent samples were included in the meta‐analysis for the FOCI and C‐FOCI.

**Results:**

We found an average KR‐20 of 0.826 for the FOCI's Symptom Checklist and an *α* of 0.882 FOCI's Symptom Severity. An average KR‐20 of 0.740 was found for the C‐FOCI's Symptom Checklist, while an average *α* of 0.794 was found for the C‐FOCI's Symptom Severity. Moderator analyses showed that the source of the coefficients (i.e., whether they were reported by the authors of the primary study or estimated by the meta‐analysts) was an important variable for the FOCI Symptom Severity, and that the focus of the study (i.e., whether it was psychometric or applied) and the sample size were relevant for the C‐FOCI Symptom Checklist.

**Conclusions:**

Considering that the FOCI and C‐FOCI are scales characterized by their brevity and ease of use, and the reliabilities obtained here, both scales are well suited for screening purposes.

## 1 INTRODUCTION

Obsessive‐compulsive disorder (OCD) is characterized by the presence of recurrent irrepressible thoughts and/or repeated behaviors. It is among the most frequent psychological disorders (Rosa‐Alcázar et al., 2008) and it is associated with functional impairment (Markarian et al., 2010) and a negative impact on quality of life (Subramaniam et al.,, 2013). The diagnostic and statistical manual of mental disorders (DSM‐5; American Psychiatric Association, 2013) included OCD under a new diagnostic category, “Obsessive‐compulsive and related disorders,” along with other related disorders such as body dysmorphic disorder, hoarding disorder, or trichotillomania.

There are more than 10 instruments available to measure OCD symptomatology in adults and children (McGuire et al., 2017). Among those, the Florida Obsessive Compulsive Inventory (FOCI; Storch et al., 2007) stands out from the rest of instruments because it is the only scale that briefly assesses both OCD symptoms and their severity in a simple, self‐report format, whereas other self‐report tests take longer to complete and/or only assess the presence of symptoms and not their severity. The FOCI comprises two scales: Symptom Checklist and Symptom Severity. Symptom Checklist is a dichotomous scale that evaluates the presence/absence of 10 common obsessions and 10 common compulsions, whereas Symptom Severity consists of 5 items that use a 5 ‐point scoring system ranging from 0 to 4, with higher scores reflecting greater severity. Furthermore, the Symptom Severity scale is not content‐dependent, which makes FOCI a more sensitive instrument to measure less common OCD symptoms and, recently, it was proven to be sensitive to clinical change as well (Veale et al., 2021). More recently, Storch et al. (2009) developed a pediatric version of the scale, the Children's Florida Obsessive Compulsive Inventory (C‐FOCI), which also includes the Symptom Checklist and Symptom Severity subscales. The first of these C‐FOCI subscales measures the presence/absence of 17 obsessions and compulsions that are frequent among young people with OCD, whereas the latter uses the same five items as the original FOCI, with small vocabulary changes. The FOCI and the C‐FOCI have been adapted and validated to other languages such as Thai (Saipanish et al., 2015), Chinese (Cao et al., 2021), and Spanish (Piqueras et al., 2016).

In its original validation study, the FOCI showed good internal consistency. Measured with the Kuder–Richardson 20 (KR‐20) formula, the reliability of the Symptom Checklist scores was 0.83 and using Cronbach's *α* for the Symptom Severity, score reliability was 0.89. Furthermore, scores showed good concurrent validity as they correlated, respectively, moderately and strongly (Symptom Checklist: *r* = 0.47, Symptom Severity: *r* = 0.89) with another OCD scale, the Yale‐Brown Obsessive‐Compulsive Scale (Y‐BOCS; Goodman et al., 1989), and also moderately correlated with the Beck Depression Inventory (Beck et al., 1961; with Symptom Checklist: *r* = 0.40, with Symptom Severity: *r* = 0.70), which can be expected to be often present in OCD (cf. Storch et al., 2007). Similarly, in the original validation study of the C‐FOCI, scores showed an acceptable internal consistency, with KR‐20 = 0.76 for Symptom Checklist and *α* = 0.79 for Severity Scale. Likewise, C‐FOCI moderately correlated with other OCD scales (Children's Yale‐Brown Obsessive Compulsive Scale [CY‐BOCS]; Scahill et al., 1997; with Symptom Checklist: *r* = 0.318, with Symptom Severity: *r* = 0.498) and scales of depression (Children's Depression Inventory Short Form; Kovacs, 1992; with Symptom Checklist: *r* = 0.351, with Symptom Severity: *r* = 0.405) and anxiety (Multidimensional Anxiety Scale for Children; March et al., 1997; with Symptom Checklist *r* = 0.607, with Symptom Severity *r* = 0.396; Storch et al., 2009).

Although in the literature we often find misleading statements such as “this test is reliable,” reliability, however, is not an inherent property of a test and it can vary each time the test is applied to a different sample of participants (Crocker & Algina, 1986; Streiner & Norman, 2008). Thus, anytime a study makes use of a scale, authors should report a reliability estimate with the data at hand (Appelbaum et al., 2018). Unfortunately, researchers often fail to report reliability estimates based on their own participants' scores, and instead it is common to find references to the reliability obtained in the original test validation study. With this practice, which is known as *reliability induction*, researchers erroneously assume that their data will offer the same reliability. Previous studies have reported reliability induction rates for psychological tests to be between 54% and 78.6% (Sánchez‐Meca et al., 2015; Vacha‐Haase et al., 1999; Whittington, 1998). Checking the reliability of the scores of newer test applications is important not only to ensure that the measure itself is reliable but also because reliability affects the effect sizes. If the test scores obtain a lower reliability, it may attenuate effect sizes that are calculated with that instrument (Vacha‐Haase et al., 2000).

Nevertheless, we can use meta‐analytic methods to obtain a representative reliability value for an instrument by integrating different reliability estimates obtained across studies. This is often referred to as reliability generalization (RG; Vacha‐Haase, 1998). Furthermore, if there is heterogeneity across reliability estimates based on the same test, an RG meta‐analysis allows us to examine whether some characteristics of the studies (i.e., moderators) might account for the variability of the reliability coefficients (Henson & Thompson, 2002; Rodriguez & Maeda, 2006; Sánchez‐Meca et al., 2013). Examples of study characteristics that can affect the reliability are the mean and variability of test scores, the target population (community, undergraduate, clinical), or whether the original version or an adaptation (to different idioms, cultures, or countries) of the test was applied.

### 1.1 Objectives

The aim of this study was to conduct an RG meta‐analysis to (a) estimate an average reliability for the FOCI and C‐FOCI by integrating all the studies that have applied the scales and reported a reliability estimate with the data at hand; (b) assess whether there is a large heterogeneity between reliability estimates for the same instrument and, if so, perform moderator analyses to identify study characteristics that account for such variability, and (c) estimate the reliability induction rate for the FOCI and C‐FOCI.

## 2 METHOD

Review methods and reporting were performed according to the *Reliability Generalization Meta‐Analysis* (REGEMA) guidelines (Sánchez‐Meca et al., 2021). The REGEMA checklist for the present meta‐analysis is available at https://osf.io/zdhvk/. We did not preregister a study protocol.

### 2.1 Participants

#### 2.1.1 Literature search

To identify studies that could be relevant for the RG meta‐analysis, we systematically searched Google Scholar, ProQuest, PsycInfo, and PubMed from 2007, the year the FOCI was published, to November 2020. We used the term “Florida Obsessive Compulsive Inventory” as the keyword to be found anywhere in the article. With this keyword we were able to identify studies that applied both the FOCI and the C‐FOCI. In addition, references cited in studies that had applied any of these scales were also consulted to identify additional studies that applied the FOCI or the C‐FOCI.

#### 2.1.2 Eligibility criteria

To be included in this RG meta‐analysis, studies had to meet three criteria: (a) to be an empirical study that applied the FOCI or the C‐FOCI to one or more samples of participants; (b) to report any reliability estimate (internal consistency, temporal stability, interrater agreement) with the data at hand or to include enough information to allow us calculate them manually; and (c) to be written in either English or Spanish language. In addition, for studies that applied the FOCI or the C‐FOCI and did not report any reliability estimate with the data at hand, the corresponding author was contacted and, if they sent any reliability estimate, the study was also included in the meta‐analysis. *N* = 1 or case series studies were excluded. Participant samples could belong to any kind of target population (community, clinical, or subclinical) and papers could be either published or unpublished (e.g., a PhD thesis dissertation).

### 2.2 Instruments

A coding form was developed to extract relevant study characteristics and reliability estimates. The coding form is available at https://osf.io/yqvhs/ and contains information about all coded variables.

### 2.3 Procedure

For the reliability estimates, we extracted the KR‐20 for the Symptom Checklist and Cronbach's *α* for the Symptom Severity subscales. Besides, for those that had a reliability estimate available, we extracted data on the following potential moderators: (a) mean and standard deviation of the scores in each scale; (b) mean and standard deviation of participants' age; (c) study sample size; (d) gender distribution (% male); (e) publication year. (f) sample ethnicity (% Caucasian); (g) target population (community, undergraduate students, clinical, or mixed); (h) location of the study (country, continent); (i) test version (original or other); (j) administration format (paper‐and‐pencil or online); (k) study aim (psychometric or applied); (l) if psychometric, the focus of the psychometric study (FOCI or other). The coding form was applied to the studies that reported any reliability estimate of the FOCI or the C‐FOCI with the data at hand. In addition, from studies that did not report reliability estimates several characteristics of the samples were also extracted: target population (clinical vs. nonclinical), mean and SD of total test scores, mean age, gender distribution (% male), and ethnicity (% of Caucasians). These variables were extracted to conduct a sensitivity analysis to explore whether the composition and variability of the inducing and reporting studies were similar. The information extraction was carried out by one author (A. S. L.).

### 2.4 Data analysis

Separate meta‐analyses were conducted for each subscale of the FOCI and C‐FOCI tests. Random‐effects models were assumed as heterogeneity among the reliability estimates was expected. The reliability coefficients were weighted by the inverse variance, using the restricted maximum likelihood method to estimate the between‐studies variance. The 95% confidence bounds were computed according to the improved method proposed by Hartung and Knapp (2001). To normalize the sampling distribution and to stabilize the sampling variances of the reliability coefficients, we applied Bonett's transformation (Bonett, 2002) to the internal consistency reliability coefficients: *L*_*i*_ = ln(1 − |*α*_*i*_|), where *α_i_* denotes the reliability coefficient of each study, ln is the natural logarithm, and *L*_*i*_ is the transformed coefficient. After the statistical integration, the *L*_*i*_ coefficients were back‐transformed to the reliability coefficient metric (*α_i_* = 1 − $e^{L_{i}}$) to facilitate interpretation and, as a sensitivity analysis, we also conducted the meta‐analyses with the untransformed coefficients (Sánchez‐Meca et al., 2013).

Heterogeneity among reliability estimates was assessed in each meta‐analysis using the *Q* statistic, the *I* ^2^ index and the calculation of 95% credibility intervals to estimate a plausible range of reliability estimates for each of the (or any future) studies (Riley et al., 2011). *I* ^2^ values of approximately 25%, 50%, and 75% have been suggested before as reflecting low, moderate, and large heterogeneity, respectively (Higgins et al., 2003), although such benchmarks were proposed in the context of clinical trials and hence they might not be applicable to the context of RG studies. If there was evidence of heterogeneity, we performed moderator analyses to identify which of the study characteristics accounted for this heterogeneity. We applied mixed‐effects meta‐regression models for continuous variables and subgroup analyses for the categorical variables with the improved method proposed by Knapp and Hartung (2003). *R* ^2^ indices were calculated to estimate the amount of variance accounted for by each moderator (López‐López et al., 2014). Furthermore, we selected the moderators that were either statistically significant or explained at least 10% of the variance (i.e., *R* ^2^ > 0.10) and made moderator models with all of their possible combinations for every scale that presented considerable heterogeneity. To compare which of the models fits best the data, we made use of an Information Theory approach based on the Akaike Information Criterion (AIC). For each model, we calculated the corrected version of the AIC (AIC_C_), recommended for small sample sizes (Symonds & Moussalli, 2011), using the maximum likelihood estimate. All statistical analyses were conducted using the metafor package in R (Viechtbauer, 2010) and the script is available at https://osf.io/65zpj/.

## 3 RESULTS

### 3.1 Study selection

**Figure 1:**
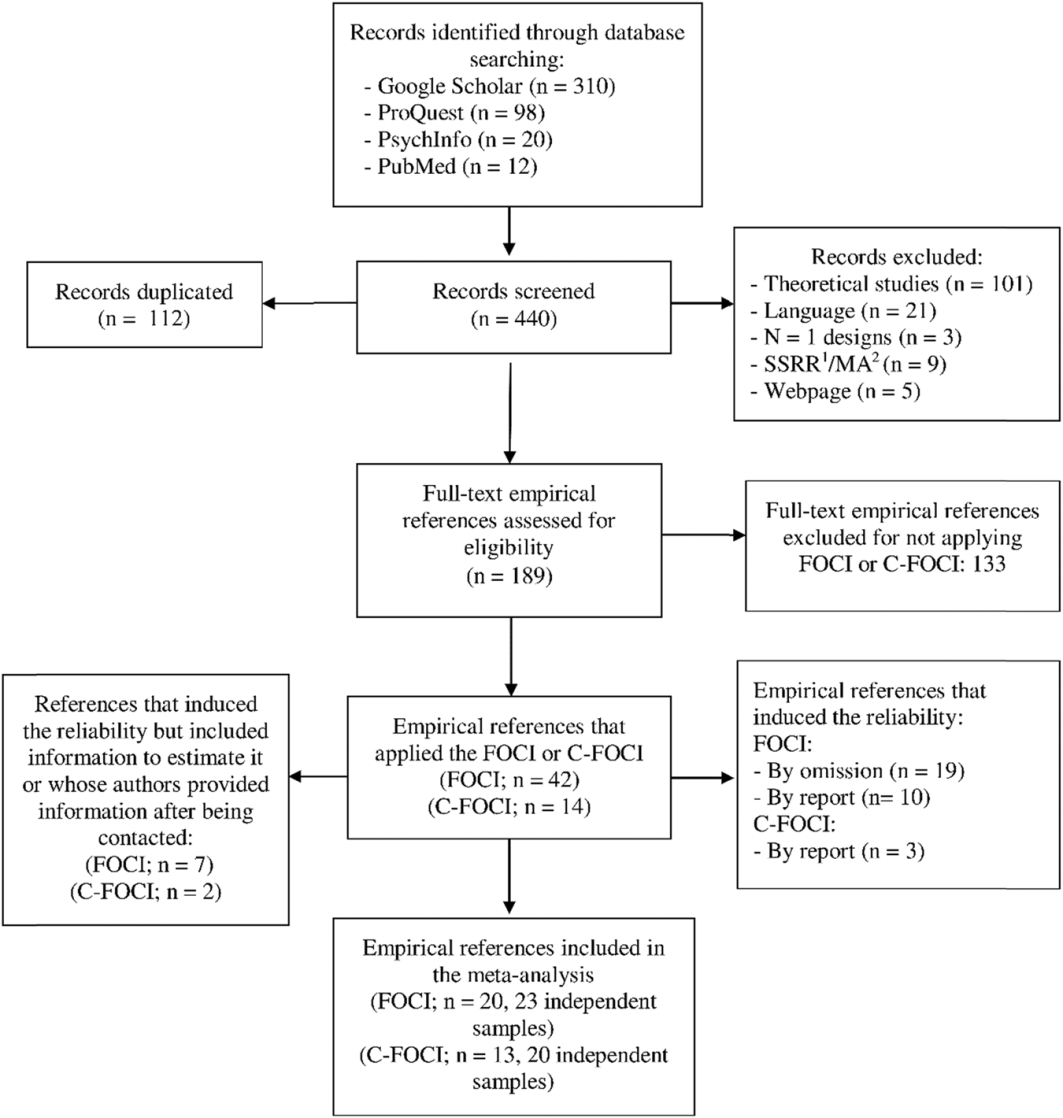
Flowchart of the study selection process

Figure 1 summarizes the entire selection process through a flowchart. A total of 440 references were initially identified. We then removed duplicated results and, based on abstract and title, excluded references that were not empirical studies. Next, we full‐text reviewed 189 references, from which 133 did not apply the FOCI or C‐FOCI. Forty‐two articles applied the FOCI, from which 13 studies (30.9%) reported some reliability estimate with the data at hand and were included in the meta‐analysis. The remaining 29 studies (69.1%) induced the reliability from previous applications of the test (by omission or by report). However, four studies reported the mean and standard deviation of the scores, enabling us to use the KR‐20 formula for the Symptom Checklist subscale, and we also obtained reliability estimates from further three studies after contacting authors via email, including those in the meta‐analysis as well and adding up to a total of 20 studies and 23 independent samples for the FOCI. Furthermore, 14 studies applied the C‐FOCI, from which 11 studies (78.6%) reported some reliability estimate with the data at hand and were included in the meta‐analysis. The remaining three studies (21.4%) induced reliability from previous applications of the test (by omission or by report). However, one study reported the mean and standard deviation of the scores, allowing us to calculate KR‐20 for the Symptom Checklist subscale, and one author sent us a reliability estimate, so these studies were also included in the meta‐analysis, adding up to a total of 13 studies with 21 independent samples. References from the studies that were included in the analyses are available at https://osf.io/6tvna/. The total sample size for the RG study of the FOCI was *N* = 4076 for the Symptom Checklist and *N* = 5898 for the Symptom Severity subscales, with range 18–986 (mean = 226; SD = 260) and 47–1224 (mean = 281; SD = 305), respectively. Most studies were carried out in North America (52.2%), followed by Asia (26.1%), Oceania (13.0%), and Europe (8.7%). The majority of studies included only people with a clinical condition (56.52%), followed by undergraduate/graduate nonclinical students (34.78%), nonundergraduate/graduate people without any known clinical condition (4.35%) and a mix of them (4.35%).

With regard to the C‐FOCI, the total sample size was *N* = 11,327 for the Symptom Checklist and *N* = 11,276 for the Symptom Severity subscales, with range 51–4293 (mean = 566; SD = 937) and 82–4293 (mean = 593; SD = 955), respectively. Most studies were carried out in Europe (65%), followed by North America (20%), South America (5%), Asia (5%), and Oceania (5%). The majority of studies included only people with a clinical condition (65%), followed by nonundergraduate/graduate people without any known clinical condition (25%) and a mix of them (10%).

Finally, concerning the reliability induction rates, 69.1% of the studies that applied the FOCI induced the reliability by omission (not making any reference to the reliability of the scale) or by report (explicitly making reference to the reliability shown in previous applications). Regarding the C‐FOCI, 21.4% of the studies that applied it induced the reliability by omission or report. The database can be found at https://osf.io/pnfwq/.

### 3.2 Mean reliability and heterogeneity

**Figure 2:**
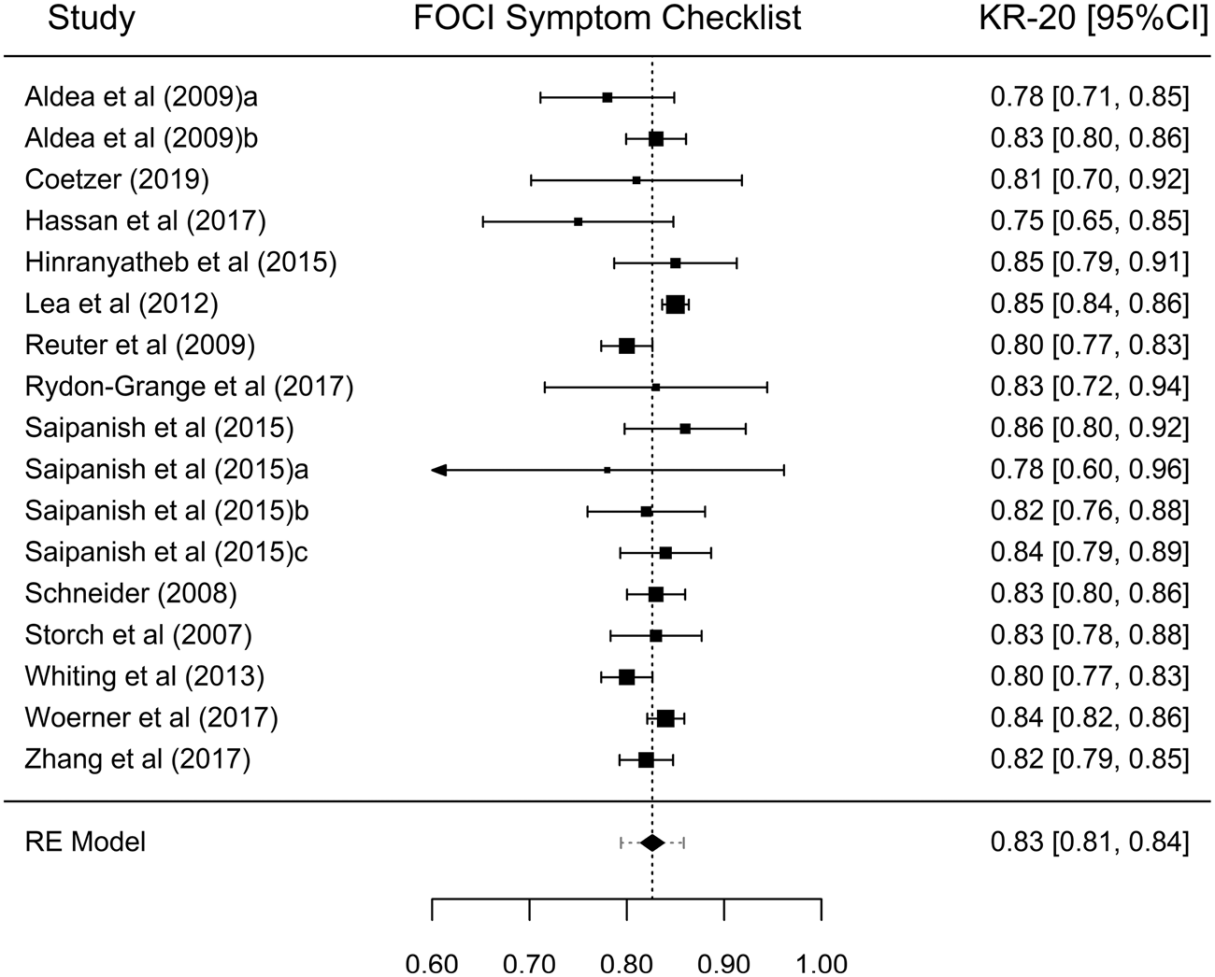
Forest plot of the KR‐20 coefficients reported for FOCI Symptom Checklist. The lines at both sides of the diamond show the limits of the 95% prediction interval. FOCI, Florida Obsessive Compulsive Inventory; KR‐20, Kuder–Richardson 20.

**Figure 3:**
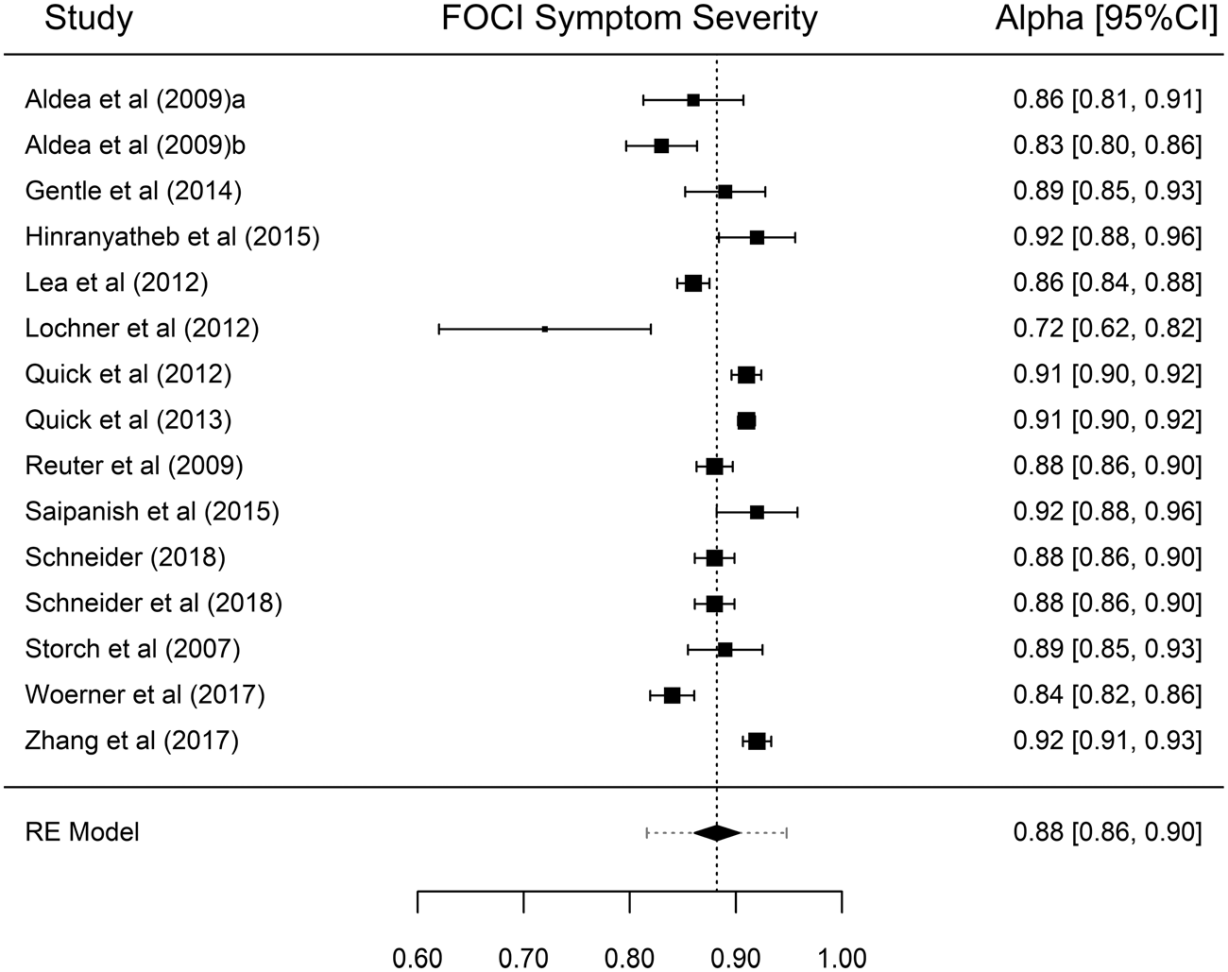
Forest plot of the *α* coefficients reported for FOCI Symptom Severity. The lines at both sides of the diamond show the limits of the 95% prediction interval. FOCI, Florida Obsessive Compulsive Inventory.

**Figure 4:**
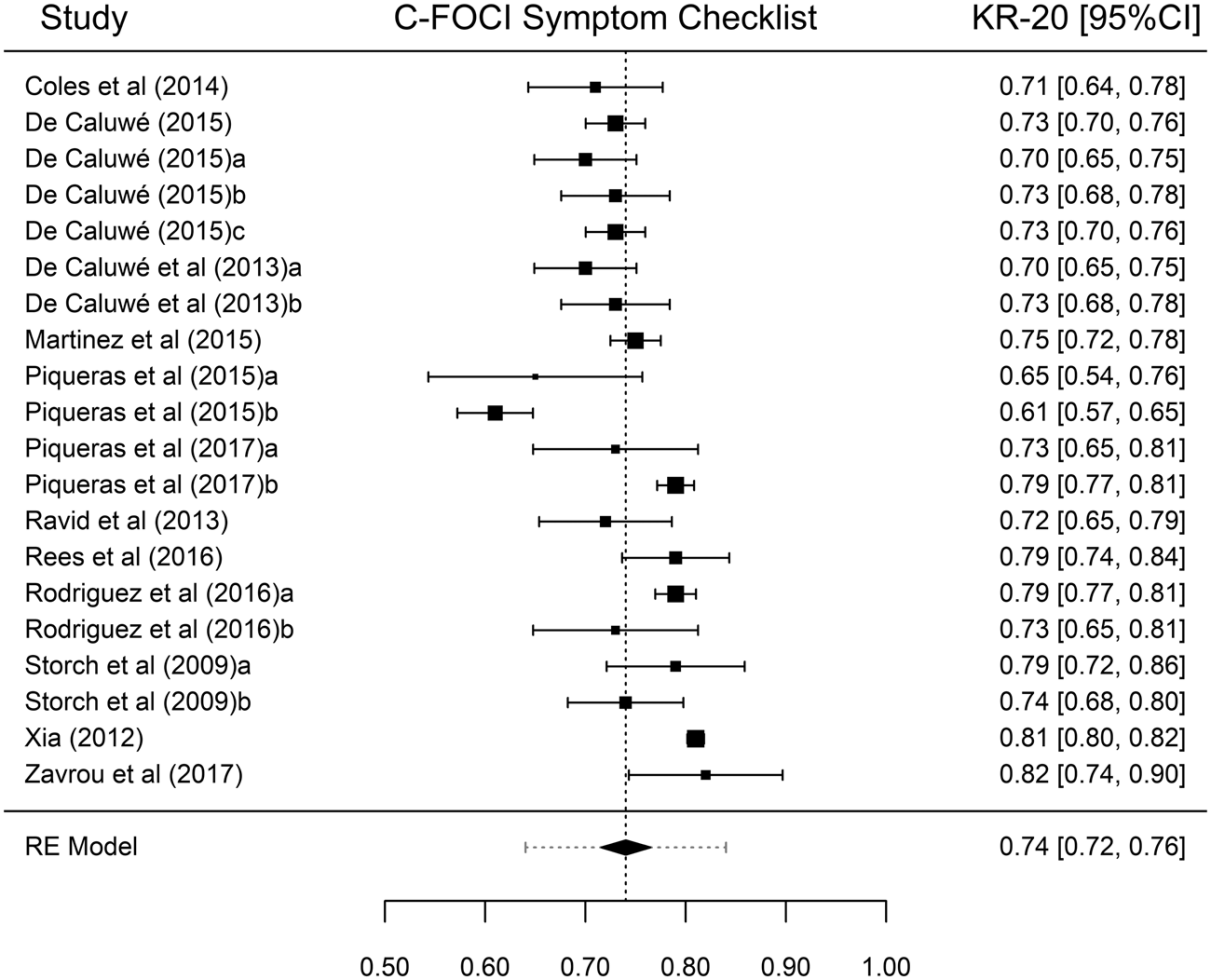
Forest plot of the KR‐20 coefficients reported for C‐FOCI Symptom Checklist. The lines at both sides of the diamond show the limits of the 95% prediction interval. C‐FOCI, Children's Florida Obsessive Compulsive Inventory; KR‐20, Kuder–Richardson 20.

**Figure 5:**
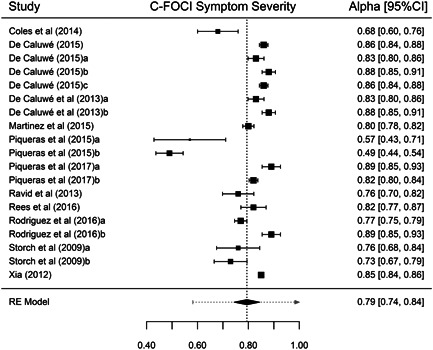
Forest plot of the *α* coefficients reported for C‐FOCI Symptom Severity. The lines at both sides of the diamond show the limits of the 95% prediction interval. C‐FOCI, Children's Florida Obsessive Compulsive Inventory.

Although our purpose was to synthesize all types of reliability coefficients, studies that applied the FOCI or the C‐FOCI only reported *α* or KR‐20 coefficients. Thus, our results were based on these types of internal consistency coefficients. Table 1 presents the main results for each of the FOCI and C‐FOCI subscales. The number of reliability coefficients extracted from the studies ranged from 15 coefficients for FOCI's Symptom Severity subscale to 20 coefficients for C‐FOCI's Symptom Checklist subscale. Because similar results were obtained using transformed or untransformed coefficients, only the results with the untransformed coefficients are presented here. Figures 2, 3, 4, 5 display forest plots of the reliability coefficients of each meta‐analyzed subscale.

**Table 1:** Mean reliability coefficients, 95% confidence and credibility/prediction intervals, and heterogeneity statistics for each subscale

Total scale	*k*	*α*_+_	95% CI		95% Cr.I		*Q*	*I* ^2^
			LL	UL	LL	UL		
FOCI Checklist	17	0.826	0.815	0.838	0.794	0.859	26.243	40.57
FOCI Severity	15	0.882	0.861	0.903	0.816	0.948	118.633*	90.04
C‐FOCI Checklist	20	0.740	0.714	0.765	0.640	0.840	201.804*	89.40
C‐FOCI Severity	19	0.794	0.744	0.844	0.582	1^a^	319.149*	98.50

Abbreviations: *α*_+_, mean coefficient *α*; *I* ^2^, heterogeneity index; *k*, number of studies; LL and UL, lower and upper limits of the 95% confidence and credibility/prediction interval for *α*_+_; *Q*, Cochran's heterogeneity *Q* statistic.

^a^ The limit estimated (1.007) exceeded the range of an *α* coefficient, therefore the value was truncated at 1.

* *p* < 0.0001.

Regarding FOCI's Symptom Checklist, a mean KR‐20 coefficient of 0.826 (95% CI [0.815, 0.838]) was found, ranging from 0.78 to 0.86. Although the *Q* statistic was not significant, there was some evidence of heterogeneity among the true reliability coefficients (*Q*(16) = 26.24, *p* = 0.064, *I* ^*2*^ = 40.57, *τ* ^*2*^ = 0.0002, 95% CrI [0.794, 0.859]). For FOCI's Symptom Severity, a mean *α* coefficient of 0.882 (95% CI [0.861, 0.903]) was found, ranging from 0.83 to 0.92; the *Q* statistic indicated heterogeneity among the true reliability coefficients (*Q*(14) = 118.63, *p* < 0.0001, *I* ^*2*^ = 90.04, *τ* ^*2*^ = 0.0008, 95% Cr.I [0.816, 0.948]). For C‐FOCI's Symptom Checklist, a mean KR‐20 coefficient of 0.74 (95% CI [0.714, 0.765]) was found, ranging from 0.61 to 0.82; the *Q* statistic reflected heterogeneity among the true reliability coefficients (*Q*(19) = 201.80, *p* < 0.0001, *I* ^*2*^ = 89.4, *τ* ^*2*^ = 0.0021, 95% Cr.I [0.640, −840]). For C‐FOCI's Symptom Severity, a mean *α* coefficient of 0.794 (95% CI [0.744, 0.844]) was found, ranging from 0.49 to 0.89; the *Q* statistic indicated heterogeneity among the true reliability coefficients (*Q*(18) = 319.15, *p* < 0.0001, *I* ^*2*^ = 98.50, *τ* ^*2*^ = 0.0097, 95% Cr.I [0.582, 1]).

### 3.3 Analysis of moderator variables

Supporting Information: Tables 1–4 available at https://osf.io/hgzmk  present the results of the meta‐regression models applied for the continuous moderators for the FOCI and the C‐FOCI. None of them exhibited a statistically significant relationship with the reliability coefficients. Nonetheless, for the C‐FOCI Symptom Checklist, there was weak evidence of sample size and standard deviation of the scores being important moderators (see Supporting Information: Table S3). In particular, the larger the sample size and the SD of test scores, the larger the reliability.

Weighted analysis of variances on the transformed reliability coefficients for each categorical moderator variable were also carried out. Full moderator analysis results are available at https://osf.io/hgzmk/. There was strong evidence of a difference when comparing the mean reliability coefficients grouped by the source from where the coefficient was obtained (reported in the paper vs. obtained from the authors upon request) for FOCI Symptom Severity (*F* (1, 13) = 9.492; *p* = 0.002; *R* ^*2*^ = 0.41; Supporting Information: Table S6). Studies that reported the coefficients exhibited higher reliability coefficients (*α*_+_ = 0.89; *k* = 13) than studies that did not report it and had to be obtained upon request (*α*_+_ = 0.80; *k* = 2). In addition, we found weak evidence of differences when comparing the mean reliability coefficients grouped by the main scale analyzed in the studies with a psychometric focus (C‐FOCI vs. other) for C‐FOCI Symptom Checklist (*F* (1, 10) = 4.681; *p* = 0.056; *R* ^*2*^ = 0.26; Supporting Information: Table S7). Studies whose psychometric focus was the C‐FOCI had higher reliability coefficients (α_+_ = 0.78; *k* = 5) than studies whose psychometric focus was another scale (α_+_ = 0.72; *k* = 7).

### 3.4 Model comparison

From all the possible moderator combinations, only the five best models (according to the AIC_C_) are presented (see Table 2). Because no moderator was statistically significant nor explained more than 10% of the variance for the FOCI Symptom Checklist, we did not perform any model comparison for this scale. For the FOCI Symptom Severity, the model that best fits our data was the one including the test adaptation and the source of the estimates as moderators, with an Akaike weight of 0.419. Models containing the source only and test adaptation and source as moderators achieved similar but slightly worse fit, all of which contained the source as a moderator. For C‐FOCI Symptom Checklist, the model with the lowest AIC_C_ was the one containing the sample size only as moderator, with an Akaike weight of 0.55, achieving slightly better fit than the model with the intercept only. Last, for C‐FOCI Symptom Severity, the best model was the one containing gender distribution (% male) as moderator, also achieving slightly better fit than the model with the intercept only.

**Table 2:** Five best‐ranked models for each of the scales according to AIC_C_

Candidate models	df	logLik	AIC_c_	${∆}_{i}$	$w_{i}$
FOCI Symptom Severity					
*Ver* + *Src*	4	2.242	7.5	0	0.419
*Src*	3	0.172	7.8	0.32	0.356
*Cnt* + *Src*	5	3.169	10.3	2.81	0.103
*Ppl* + *Src*	5	3.015	10.6	3.12	0.088
*Ver*	3	−2.161	12.5	4.99	0.035
C‐FOCI Symptom Checklist					
*Siz*	3	6.422	−5.3	0	0.550
*Int*	2	4.441	−4.2	1.17	0.307
*Frm*	4	6.449	−2.2	3.11	0.116
*Frm* + *Siz*	5	6.582	1.1	6.47	0.022
*Cnt* a	6	7.327	3.8	9.15	0.006
C‐FOCI Symptom Severity					
*Gen*	3	−7.895	23.8	0	‐
*Int*	2	−10.72	26.2	2.4	‐

Abbreviations: *Cnt*, continent; *Frm*, administration format; *Gen*, gender distribution (% males); *Int*, intercept only; *Ppl*, target population; *Siz*, sample size; *Src*, estimates source; *Ver*, test version.

^a^ Although Continent exhibited an *R* ^2^ = 0.09, it was also included.

## 4 DISCUSSION

We performed an RG meta‐analysis to characterize how reliability of the FOCI and the C‐FOCI varies across studies. The FOCI showed an average reliability of 0.826 for the Symptom Checklist subscale and 0.882 for the Symptom Severity subscale, whereas the C‐FOCI showed an average reliability of 0.740 for the Symptom Checklist subscale and 0.794 for the Symptom Severity subscale. Acording to Cicchetti (1994), these values can be considered as good (FOCI) and fair (C‐FOCI) reliability coefficients, respectively. If we compare these values to those obtained in other RG meta‐analyses of other OCD scales such as the Y‐BOCS, the original Padua Inventory (PI) and their two revisions (the Padua Inventory Revision [PI–R], and the Padua Inventory–Washington State University Revision [PI–WSUR]), the Maudsley Obsessional Compulsive Inventory and the Dimensional Obsessive‐Compulsive Scale (López Nicolás et al., 2021; López‐Pina et al., 2015; Núñez‐Núñez et al., 2022; Rubio‐Aparicio et al., 2020; Sánchez‐Meca et al., 2011, 2017), we see that they have similar or slightly better psychometric properties. Nonetheless, we want to again emphasize that the FOCI and C‐FOCI are the only OCD scales that briefly examine both OCD symptoms and their severity, while also being brief, simple, and self‐reported. The average reliabilities obtained here are above 0.80 for the FOCI and 0.70 for the C‐FOCI. Considering the guidelines given by Nunnally and Bernstein (1994), where 0.70 is considered an acceptable standard for instruments used in basic research and quick assessments, 0.80 is suitable for research comparing the scores of different groups, and 0.90 is needed for clinical settings where diagnostics or other important decisions are made, the average reliabilities obtained in the present study make both scales seem especially relevant to be applied on community population for screening purposes of OCD symptoms and, additionally for the FOCI, also to be applied when conducting a research involving group comparisons.

A large heterogeneity among coefficients was found for both FOCI subscales and in the C‐FOCI Symptom Checklist subscale so we performed moderator analyses to identify which study characteristics could be explaining this variability. For continuous moderators, we found that none of them were statistically associated with the reliability coefficients, although there was some evidence that sample size and standard deviation of the scores accounted for a substantial part of the variability among the reliability estimates. The standard deviation of the scores has been previously found to be a source of systematic variation of the reliability coefficients (Botella et al., 2010). Thus, what might be surprising here is that, even though in many cases the standard deviation explained a considerable portion of the variance, it did not reach statistical significance. Considering the scarce number of studies that have reported the standard deviation of the test scores, this lack of statistical significance might be due to a low statistical power. Another aspect to take into consideration is the fact that most studies only applied the FOCI or C‐FOCI to a particular sample (either people with a clinical condition or without one, but not both). This might be artificially restricting the standard deviations, making them very similar across studies and, therefore, preventing their potential role as a moderator variable. For the categorical moderators, we found that, in the FOCI's Symptom Severity subscale, the source from where the coefficient was obtained (either reported in the paper or requested to the authors) was a significant moderator. Notably, studies that reported the coefficients showed higher reliability compared to studies where our team requested them from the authors. This finding can be interpreted as indicating the existence of reporting bias of the reliability coefficients, such that studies are less likely to report the reliability coefficient obtained with the data at hand when the observed value is small. We also found that, in C‐FOCI's Symptom Checklist, studies whose psychometric focus was the C‐FOCI showed higher reliability coefficients than studies whose psychometric focus was another scale. Lastly, another remarkable result was that the administration format was not a significant moderator in any of the scales, that is, there was no difference between the reliability estimates when they were obtained in a pencil and paper format compared to when they were obtained online. Thus, both the FOCI and the C‐FOCI might be used as online screening tools to assess OCD symptoms easily and quickly.

Model comparison also showed that, for the C‐FOCI Symptom Checklist, sample size might be a relevant moderator of the reliability coefficients, with a positive association between sample size and the magnitude of the reliability coefficients. Last, for the C‐FOCI Symptom Severity, gender might also be important as a moderator, since the model containing gender achieved the best fit. Specifically, there appears to be a negative trend toward the percentage of males in the sample and the reliability scores.

Finally, we also calculated the reliability induction rate for both scales. For the FOCI, we found a rather high induction rate (69.05% of the studies induced the reliability). This finding is in line with previous appraisals of this practice (Sánchez‐Meca et al., 2015; Vacha‐Haase et al., 1999; Whittington, 1998). Considering that this scale is rather recent, this result suggests that the practice of inducing reliability of test scores from previous studies is still extended. On the other hand, only 21.43% of the studies that applied the C‐FOCI induced the reliability by omission or report. This considerably lower rate of induction (compared to the FOCI and other scales) might be due to the fact that half of the studies that applied the C‐FOCI had a psychometric focus (by contrast, only 5.25% of the studies that applied the FOCI had a psychometric focus), and it is reasonable to expect that psychometric studies of a test will calculate and report reliability coefficients.

### 4.1 Limitations

Although many studies have applied the FOCI or C‐FOCI in their research, the number of studies that have reported a reliability estimate with the data at hand is considerably smaller. This, together with the fact that missing data on potential moderator variables was common, may have limited the results of the moderator analyses. Another limitation was the failure of authors to report important study characteristics whose potential influence on the reliability estimates can be investigated in an RG meta‐analysis. In particular, many studies did not report the mean and standard deviation of test scores, two very important moderators in the context of RG studies.

On another note, this RG meta‐analysis was based on Cronbach's *α* (and KR‐20, a special case of *α*). Because Cronbach's *α* does not require multiple test administrations to estimate the reliability of a scale, it is the most commonly reported reliability coefficient (Hogan et al., 2000; Scherer & Teo, 2020). However, using Cronbach's *α* as a measure of reliability presents some drawbacks. First, this coefficient is not invariant to the numbers of items in the scale, larger number of items will inflate Cronbach's *α* (Tavakol & Dennick, 2011). Second, it relies on some assumptions that are usually not met in practice (*τ*‐equivalence, uncorrelated item errors, unidimensionality, etc.), resulting in an over or underestimation of the reliability (McNeish, 2018). For this reason, Cronbach's *α* is being criticized and it is advised that future primary studies should, instead, make use of composite reliability measures such as the omega coefficients or measures of maximal reliability such as the coefficient H, allowing future RG meta‐analyses to be based on those coefficients (Scherer & Teo, 2020). Nonetheless, studies published to date have seldom used these alternative reliability coefficients, so an RG meta‐analysis based on them is not yet feasible for most measurement instruments.

## 5 CONCLUSIONS

Our findings show that the FOCI and C‐FOCI instruments present, on average, good and acceptable reliability values, respectively. Given the brevity and ease of use of these two scales and that they are able to simultaneously assess OCD symptoms and their severity, both tests can be recommended for the screening of OCD symptoms. For the FOCI, we found a large rate of reliability induction, showing how this practice is still extended even in recent empirical studies using psychometric tests. For the C‐FOCI, we found a much lower rate of reliability induction, although this result is probably accounted by the fact that half of the studies that applied the C‐FOCI had a psychometric focus. Thus, it is still necessary to emphasize that studies that make use of a psychometric scale should estimate reliability with the data at hand, rather than insisting on the erroneous practice of reporting estimates obtained in previous applications of the instrument.

## CONFLICT OF INTEREST

The authors declare no conflict of interest.

### PEER REVIEW

The peer review history for this article is available at https://publons.com/publon/10.1002/jclp.23416

## Supporting information

Supplementary Information

## Data Availability

The data that support the findings of this study are openly available at https://osf.io/zxn2k/.
