# Supplementary material for: Florida Obsessive‐Compulsive Inventory and Children's Florida Obsessive Compulsive Inventory: A reliability generalization meta‐analysis
Source: J Clin Psychol. 2022 Jul 18;79(1):28–42. doi: 10.1002/jclp.23416 (PMC10084361; doi:10.1002/jclp.23416)
Supplement: Supplementary file 1 — Supplementary Information [file JCLP-79-28-s001.DOCX]

Supplementary data to this article can be found online at https://osf.io/zxn2k/
